# Supplementary material for: Genetic comparisons of interleukin-17 reveal a framework for complex signaling evolution
Source: bioRxiv. 2026 Apr 14:2026.04.13.718218. Preprint. [Version 1] doi: 10.64898/2026.04.13.718218 (PMC13105070; doi:10.64898/2026.04.13.718218)
Supplement: Supplement 3 [file media-3.zip › Supplemental files for paper/ERC/IL17xIL17_comparisons/IL17F_IL25.pdf]

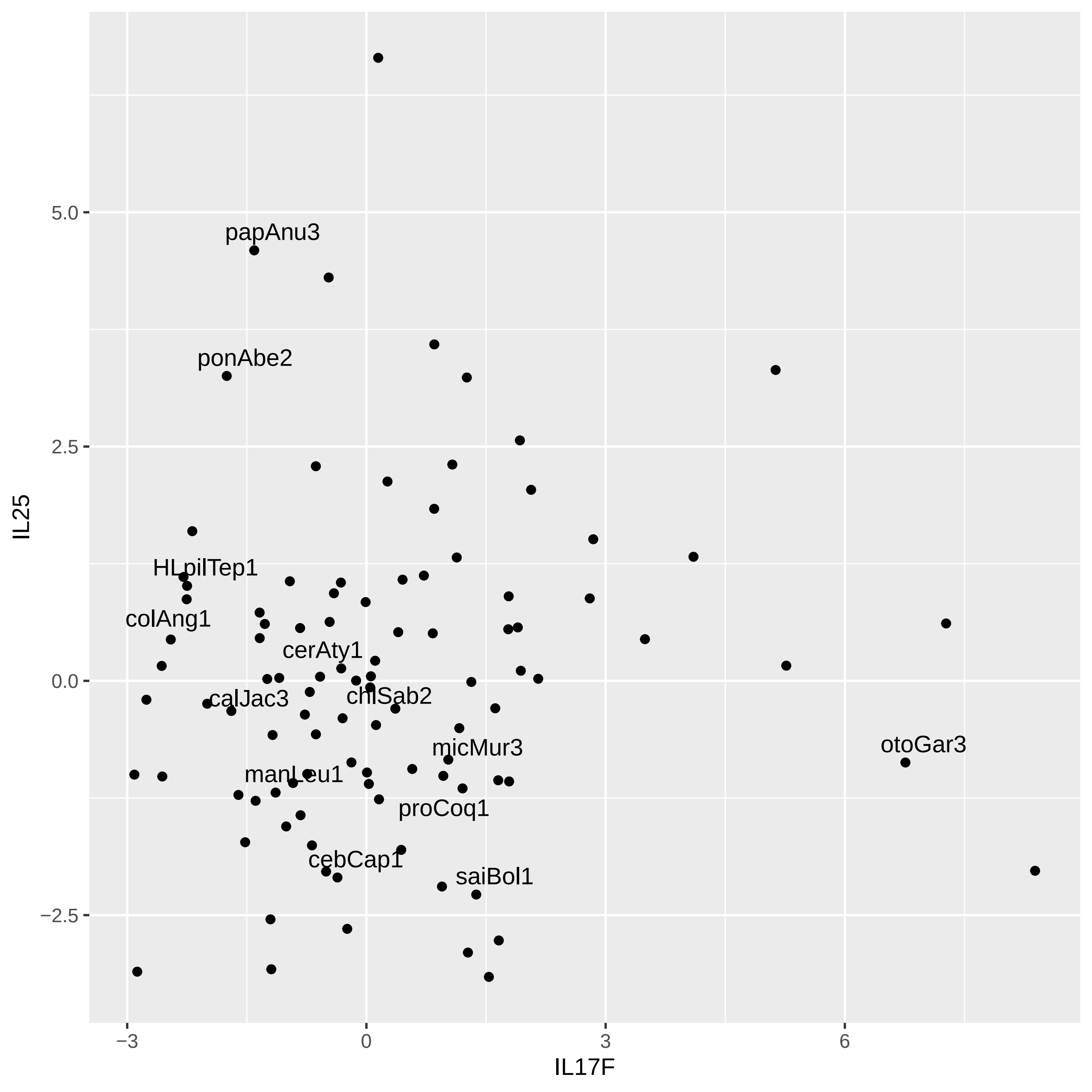

papAnu3
ponAbe2
HLpilTep1
colAng1
cerAty1
calJac3
chlSab2
micMur3
otoGar3
manLeu1
proCoq1
cebCap1
saiBol1
5.0
2.5
0.0
−2.5
IL25
−3
0
3
6
IL17F
